# Neurosurgical experience of managing optic pathway gliomas

**DOI:** 10.1007/s00381-021-05060-8

**Published:** 2021-02-03

**Authors:** Ciaran Scott Hill, Mehdi Khan, Kim Phipps, Katherine Green, Darren Hargrave, Kristian Aquilina

**Affiliations:** 1 Department of Neurosurgery, Great Ormond Street Hospital, London, UK; 2 UCL Cancer Institute, University College London, London, UK; 3 University College London Medical School, London, UK; 4 Department of Pediatric Oncology, Great Ormond Street Hospital London, London, UK; 5 Department of Pediatric Oncology, UCL Great Ormond Street Institute of Child Health and Great Ormond Street Hospital, London, UK

**Keywords:** Optic, Visual, Pathway, Glioma, Management

## Abstract

**Background:**

Optic pathway gliomas (OPGs), also known as visual pathway gliomas, are debilitating tumors that account for 3–5% of all pediatric brain tumors. They are most commonly WHO grade 1 pilocytic astrocytomas and frequently occur in patients with neurofibromatosis type 1. The location of these tumors results in visual loss and blindness, endocrine and hypothalamic dysfunction, hydrocephalus, and premature death. Their involvement of the visual pathways and proximity to other eloquent brain structures typically precludes complete resection or optimal radiation dosing without incurring significant neurological injury. There are various surgical interventions that can be performed in relation to these lesions including biopsy, cerebrospinal fluid diversion, and partial or radical resection, but their role is a source of debate. This study catalogues our surgical experience and patient outcomes in order to support decision-making in this challenging pathology.

**Methods:**

A retrospective review of all cases of OPGs treated in a single center from July 1990 to July 2020. Data was collected on patient demographics, radiographic findings, pathology, and management including surgical interventions. Outcome data included survival, visual function, endocrine, and hypothalamic dysfunction.

**Results:**

One hundred twenty-one patients with OPG were identified, and 50 of these patients underwent a total of 104 surgical procedures. These included biopsy (31), subtotal or gross total resection (20 operations in 17 patients), cyst drainage (17), Ommaya reservoir insertion (9), or cerebrospinal fluid diversion (27). During the study period, there was 6% overall mortality, 18% hypothalamic dysfunction, 20% endocrine dysfunction, and 42% had some cognitive dysfunction. At diagnosis 75% of patients had good or moderate visual function in at least one eye, and overall, this improved to 83% at the end of the study period. In comparison the worst eye had good or moderate visual function in 56%, and this reduced to 53%. Baseline and final visual function were poorer in patients who had a surgical resection, but improvements in vision were still found—particularly in the best eye.

**Discussion/conclusion:**

OPG are debilitating childhood tumor that have lifelong consequences in terms of visual function and endocrinopathies/hypothalamic dysfunction; this can result in substantial patient morbidity. Decisions regarding management and the role of surgery in this condition are challenging and include cerebrospinal fluid diversion, biopsy, and in highly select cases cystic decompression or surgical resection. In this paper, we review our own experience, outcomes, and surgical philosophy.

## Introduction

Optic pathway gliomas (OPGs) are debilitating tumors that account for 3–5% of all pediatric brain tumors. Histologically they are frequently the World Health Organization (WHO) grade 1 juvenile pilocytic astrocytomas (JPA), with a smaller proportion being pilomyxoid astrocytomas (PXA) [1, 2]. They are common in patients with neurofibromatosis type 1 (NF1), with up to 20% of these patients developing an OPG at a mean age of 4.5–5 years old [3]. The implicated genetic culprit is typically related to aberrations in the RAS/Raf/MAPK pathway such as loss of NF1 product (neurofibromin)-related suppression of RAS, B-Raf activation by the KIAA1549-BRAF fusion product, or less commonly as a function of BRAF-V600E point mutation [3–6]. Such mutations are unusual among gliomas in that they are sufficient to induce tumorigenesis in isolation [7].

OPGs can occur anywhere along the optic pathway but most commonly arise at the chiasmatic-hypothalamic region [8]. The location of these tumors means that up to half may present initially with visual dysfunction or progress to blindness. Also common are endocrine and hypothalamic dysfunction and hydrocephalus. Many of the sequelae of OPG are lifelong and can result in substantial patient morbidity [9–15]. Prognostication is unreliable, but the female sex, a location posterior to the optic chiasm, and presentation at an early (<2 years) or late age (>10 years) seem to have more aggressive trajectories, while association with NF 1 is generally considered a favorable prognostic marker [16–18]. The effect of an OPG on overall survival is variable, and tumor stabilization, progression, or regression can all occur unpredictably. Key aspects of their behavior including their natural history are incompletely understood [19].

The complexity of symptomatology and the close relationship to key structures make the treatment of OPG highly challenging. Their involvement of the visual pathways and proximity to other eloquent brain structures typically precludes complete resection or optimal radiation dosing without incurring significant neurological injury. Therefore, OPG management is necessarily highly individualized. Many patients undergo a period of observation with serial imaging unless there is progressive tumor growth or visual symptoms. Chemotherapy, radiotherapy, and surgical intervention are all options. Decision-making is multidisciplinary as standard and may involve neurosurgeons, neuro-oncologists, radiation oncologists, endocrinologists, ophthalmologists, pathologists, neuropsychology, pediatricians, geneticists, and a host of allied health professionals. This multifaceted care underlines the complexity of the disease. Chemotherapy with a “Packer” regimen of carboplatin and vincristine is often the first line, although alternatives such as single agent vinblastine, TPCV (thioguanine, procarbazine, lomustine, vincristine), and bevacizumab containing regimens may be used [20, 21]. Radiotherapy is typically avoided where possible in younger children and due to risks of toxicity, but it is an option in the setting of refractory disease with progressive visual loss occurring despite systemic therapy [22–24]. More recently clinical trials of oral targeted small molecule MAPK inhibitors such as dabrafenib and selumetinib have shown preliminary activity in pediatric low-grade glioma, and randomized upfront studies of these novel therapies versus standard chemotherapy are ongoing or planned [25, 26].

The perceived role of surgery in OPGs has varied over time, and across published series and consensus remains elusive. The are several potential surgical interventions that can be performed in relation to these lesions including biopsy, cerebrospinal fluid diversion, cyst drainage, and partial or complete resection. However, their role continues to be a source of debate and controversy [27, 28]. This study aims to catalogue our surgical experience and patient outcomes in OPGs to help support decision.

## Methods

A retrospective review of all OPGs treated between July 1990 and July 2020 at Great Ormond Street Hospital (GOSH) was carried out. GOSH is a dedicated pediatric tertiary center in London, UK. Electronic patient records and case notes were reviewed, and data was collected on demographics including age at presentation, gender, ethnicity, presenting features, radiographic and histological findings, NF1 status, and interventions including chemotherapy, radiotherapy, and surgery. Outcome data was collated on survival, visual function, endocrine, and hypothalamic dysfunction. As this was a retrospective observational study, there was no *a priori* specified treatment strategy.

## Results

One hundred twenty-two patients with OPG were identified over a 30-year period (July 1990 to July 2020) at GOSH. Fifty-one of these patients had undergone at least one related surgical intervention with 10 of these being resections.

The summary demographics of these patients are presented in Table 1.

**Table 1:** Summary demographic details

	All cases	Patients who underwent resection	Patients who underwent any non-resection surgical intervention	Not operated
Total number	121	17	33	71
Age at diagnosis (mean)	5.3 years average (0–16.2 range)	1.8 years average (0.5–7.5 range)	5.7 years average (0–14.8 range)	5.8 years average (0.3–16.2 range)
Ethnicity	65% White (79/121) 12% Asian (15/121) 13% Middle eastern (16/121) 9% Black (11/121)	59% White (10/17) 12% Asian (2/17) 18% Middle eastern (3/17) 12% Black (2/17)	58% White (19/33) 12% Asian (4/33) 15% Middle eastern (5/33) 15% Black (5/33)	70% White (50/71) 13% Asian (9/71) 11% Middle eastern (8/71) 6% Black (4/71)
Sex	51% female (62/121) 49% male (59/121)	41% female (7/17) 59% male (10/17)	58% female (19/33) 42% male (14/33)	51% female (36/71) 49% male (35/71)
Neurofibromatosis 1	38% (46/121)	6% (1/17)	24% (8/33)	52% (37/71)
Past medical history	3% asthma (3/121) <1% acute lymphocytic leukemia (1/121) <1% congenital cardiac anomaly (1/121) <1% spastic diplegia (1/121) <1% recurrent ear infections (1/121)		3% asthma (1/33) 3% recurrent ear infections (1/33)	3% asthma (2/71) 1% acute lymphocytic leukemia (1/71) 1% congenital cardiac anomaly (1/71) 1% spastic diplegia (1/71)
Primary presenting symptom	45% visual symptoms (54/121) 17% screening/investigations for NF1 (21/121) 12% headache (14/121) 7% seizure (9/121) 7% hypothalamic symptoms or signs including precocious puberty or diencephalic syndrome (8/121) 6% motor symptoms/focal neurology (7/121) 3% failure to thrive (4/121) 2% other (2/121) 2% unknown (2/121)	65% visual symptoms (11/17) 12% headache (2/17) 18% motor symptoms/focal neurology (3/17) 6% failure to thrive (1/17)	30% visual symptoms (10/33) 15% screening/investigations for NF1 (5/33) 22% headache (7/33) 9% seizure (3/33) 3% hypothalamic symptoms or signs including precocious puberty or diencephalic syndrome (1/33) 9% motor symptoms/focal neurology (3/33) 9% failure to thrive (3/33) 3% other (1/33) 3% unknown (1/33)	47% visual symptoms (33/71) 23% Screening/investigations for NF1 (16/71) 7% headache (5/71) 9% seizure (6/71) 10% hypothalamic symptoms or signs including precocious puberty or diencephalic syndrome (7/71) 3% motor symptoms/focal neurology (2/71) 0% failure to thrive (0/71) 1% other (1/71) 1% unknown (1/71)
Histological diagnosis	70% unavailable (85/121)	59% pilocytic astrocytoma (10/17) 6% pilomyxoid astrocytoma (1/17) 29% low-grade glioma–unspecified (5/17) 6% ganglioglioma (1/17)	42% unavailable (14/33) Of those available: 74% pilocytic astrocytoma (14/19) 26% low-grade glioma–unspecified (5/19) 5% high-grade glioma –unspecified (1/19)	100% unavailable (71/71)
Dodge stage	10% A (12/121) 34% B (41/121) 56% C (68/121)	29% A (5/17) 29% B (5/17) 41% C (7/17)	9% A (3/33) 67% B (22/33) 24% C (8/33)	6% A (4/71) 20% B (14/71) 75% C (53/71)
Hypothalamic involvement				
Radiological Clinical	53% (64/121) 18% (22/121)	52% (9/17) 41% (7/17)	82% (27/33) 18% (6/33)	39% (28/71) 13% (9/71)

Details regarding treatments including surgery are presented in Table 2.

**Table 2:** Summary treatment details

Number of patients operated	41% (50/121)
Total number of surgeries performed	104 on 50 patients
Number of operative episodes per patient *(each episode may include >1 procedure e.g., subtotal resection and placement of Ommaya reservoir under one anesthetic = 1)*	59% nil (71/121) 41% one (50/121) 16% two (19/121) 6% three (7/121)
Patients with number of separately classified procedures *(different procedures may have occurred under a single operative episode, e.g., subtotal resection and placement of Ommaya reservoir = 2)*	59% nil (71/121) 22% one (23/104) 14% two (14/104) 5% three (5/104) 4% four (4/104) 2% five (2/104) 2% six (2/104)
Types of operations performed	30% biopsy (31/104) 25% ventriculoperitoneal shunt (26/104) 16% cyst drainage (17/104) 15% subtotal resection (16/104) 9% Ommaya reservoir (9/104) 4% complete resection (4/104) 1% endoscopic third ventriculostomy (1/104)
Surgical complication	6% shunt failure (6/104) 1% bleeding into tumor (1/104)
Chemotherapy	% (65/121)
Chemotherapy complications	65% none (42/65) 10% minor (7/65) 25% major/requiring change in regimen (16/65)
Radiotherapy	14% (17/121)
Radiotherapy complication	12% (2/17)
Proton beam therapy	8% (4/121)

Details regarding outcomes are presented in Table 3

**Table 3:** Summary outcome details

	All patients	Patients who underwent resection	Patients who underwent any non-resection surgical intervention	Patients who did not have surgery
Endocrine status				
No deficit Single hormone deficiency Multiple hormone deficiency	80% (97/121) 17% (20/121) 3% (/121)	65% (11/17) 12% (2/17) 24% (4/17)	88% (29/33) 12% (4/33) 0% (0/33)	80% (57/71) 20% (14/71) 0% (0/71)
Cognitive difficulty				
No dysfunction Yes Unable to assess	82% (99/121) 17% (50/121) 1% (1/121)	77% (13/17) 23% (4/17) 0% (0/17)	82% (27/33) 12% (4/33) 3% (1/33)	83% (59/71) 17% (12/71) 0% (0/71)
Recurrence				
No Yes	59% (71/121) 41% (50/121)	18% (3/17) 82% (14/17)	39% (13/33) 60% (20/33)	77% (55/71) 23% (16/71)
Mortality within study period	5% (6/121)	6% (1/17)	9% (3/33)	3% (2/71)

**Fig. 1:**
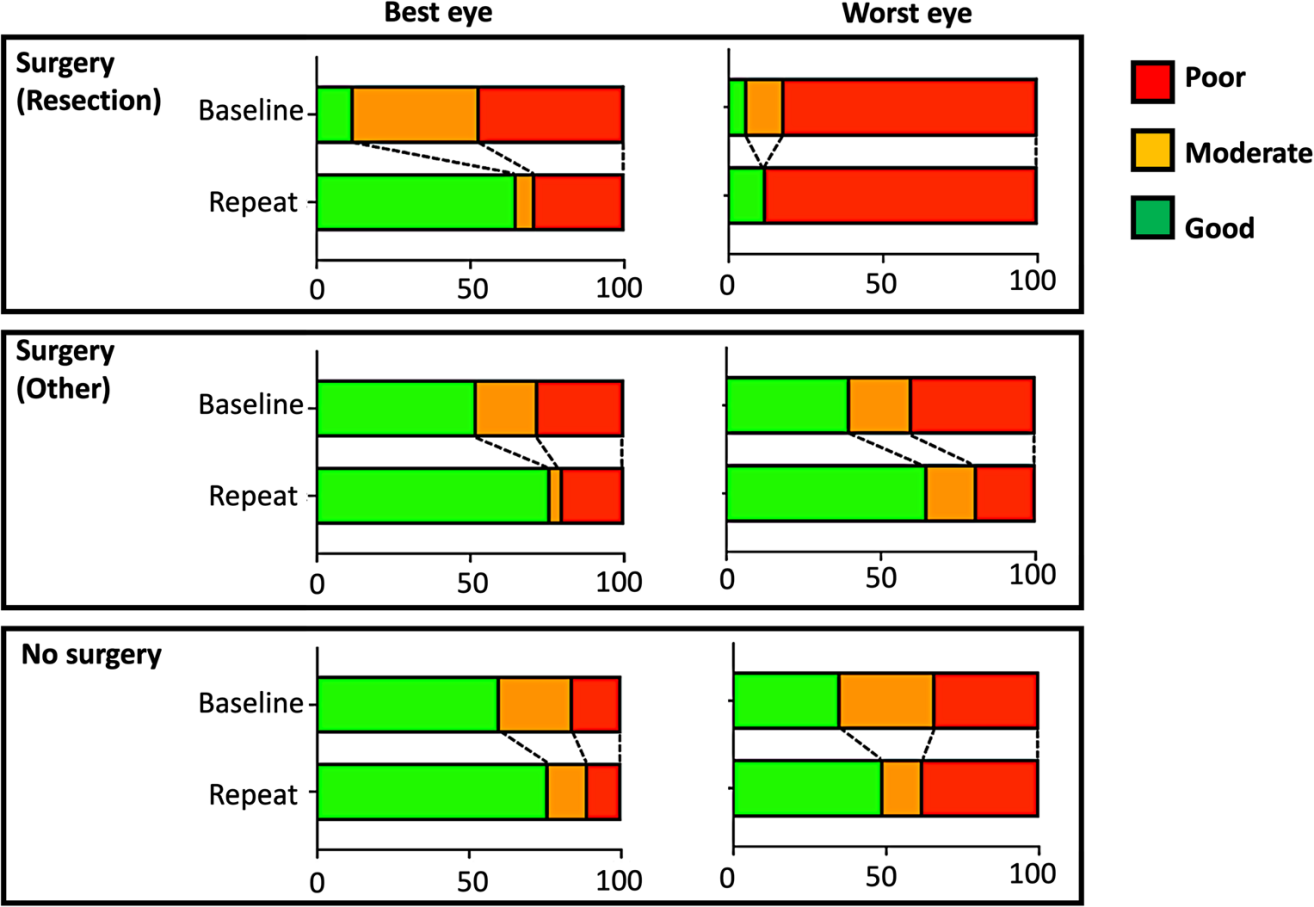
Summary of baseline and repeat visual outcomes classified by treatment group (resective surgery, non-resective surgery, or no surgery) and stratified by the eye with best and worst vision. The initial baseline visual assessment refers to the earliest on record, and the repeat the most recent on record

Details regarding visual outcomes are presented in Fig. 1.

The details of the 17 resection surgeries undertaken are presented in Table 4.

**Table 4:** Details of patients who underwent resection surgery

Patient number	Age at diagnosis	Pathology	Dodge (plan criteria)	Surgery undertaken (each discrete episode is numbered)	Outcomes
1	2.5	Pilocytic astrocytoma	2 (2a)	1. Subtotal resection and ventriculoperitoneal shunt 2. Ommaya reservoir 3. Redo subtotal resection	Visual improvement in one eye Multiple hormone deficiency Cognitive/behavioral difficulties Clinical hypothalamic dysfunction Surgical complication - bleed into tumor
2	4.9	Pilocytic astrocytoma (KIAA1549:BRAF fusion)	2 (1cL2CL)	1. Subtotal resection	No change in vision No endocrine dysfunction No hypothalamic dysfunction Cognitive/behavioral difficulties No surgical complications
3	6.9	Pilocytic astrocytoma	2 (2a)	1. Subtotal resection	No change in vision Multiple hormone deficiency Clinical hypothalamic dysfunction No cognitive/behavioral difficulties No surgical complications
4	3.3	Pilocytic astrocytoma (KIAA1549:BR AF fusion)	3 (1b1c2a3)	1. Subtotal resection 2. Redo subtotal resection and ventriculoperitoneal shunt 3. Cyst drainage and insertion of Ommaya	No change in vision No hormone deficiency Clinical hypothalamic dysfunction No cognitive/behavioral difficulties No surgical complications
5	3.5	Low-grade glioma (unspecified)	1 (1aL)	1. Subtotal resection	Visual improvement in one eye No hormone deficiency No hypothalamic dysfunction No cognitive/behavioral difficulties No surgical complications
6	3.8	Pilocytic astrocytoma	1 (1aR)	1. Gross total resection	No change in vision No hormone deficiency No hypothalamic dysfunction Cognitive/behavioral difficulties No surgical complications
7	2	Pilocytic astrocytoma	1 (1aR)	1. Gross total resection	Visual improvement in one eye No hormone deficiency No hypothalamic dysfunction No cognitive/behavioral difficulties No surgical complications
8	1.7	Low-grade glioma (unspecified)	2 (1aR2BR)	1. Subtotal resection 2. Insertion of Ommaya reservoir 3. Redo subtotal resection and insertion, cyst drainage and insertion of second Ommaya reservoir	Visual improvement in one eye Multiple hormone deficiencies No hypothalamic dysfunction No cognitive/behavioral difficulties No surgical complications Death aged 3yrs 11 months
9	1.9	Pilocytic astrocytoma	1 (1aR1cR)	1. Gross total resection	No change in vision No hormone deficiency No hypothalamic dysfunction No cognitive/behavioral difficulties No surgical complications
10	2.3	Pilocytic astrocytoma (BRAF wild type)	1 (1aR)	1. Biopsy 2. Gross total resection	No change in vision No hormone deficiency No hypothalamic dysfunction No cognitive/behavioral difficulties No surgical complications
11	1.2	Pilomyxoid astrocytoma (BRAF V600E mutation)	3 (1bR2bR3b)	1. Biopsy 2. Subtotal resection	Visual deterioration in both eyes No hormone deficiency Hypothalamic dysfunction No cognitive/behavioral difficulties No surgical complications
12	0.7	Low-grade glioma (unspecified)	3 (2bR3R)	1. Cyst drainage 2. Subtotal resection and ventriculoperitoneal shunt	No vision information available Multiple hormone deficiency Hypothalamic dysfunction No cognitive/behavioral difficulties No surgical complications
13	5.8	Pilocytic astrocytoma	3 (2b3B)	1. Biopsy and ventriculoperitoneal shunt 2. Subtotal resection, cyst drainage, and ventriculoperitoneal shunt revision	No change in vision Single hormone deficiency No hypothalamic dysfunction Cognitive/behavioral difficulties Surgical complication – shunt failure
14	0.5	Pilocytic astrocytoma (BRAF wild type)	3 (1c2b3b)	1. Biopsy and ventriculoperitoneal shunt 2. Subtotal resection	No change in vision No hormone deficiency No hypothalamic dysfunction No cognitive/behavioral difficulties No surgical complications
15	0.5	Ganglioglioma	3 (3L)	1. Biopsy and ventriculoperitoneal shunt 2. Subtotal resection and cyst drainage	Visual improvement in one eye No hormone deficiency Hypothalamic dysfunction No cognitive/behavioral difficulties No surgical complication
16	1.1	Low-grade glioma (unspecified)	2 (1cR2bR)	1. Ventriculoperitoneal shunt 2. Subtotal resection	Visual improvement in both eyes No hormone deficiency Hypothalamic dysfunction No cognitive/behavioral difficulties No surgical complications
17	2.4	Low-grade glioma (unspecified)	3 (1bB1cB2a3B4B)	1. Ventriculoperitoneal shunt 2. Subtotal resection and ventriculoperitoneal shunt revision 3. Redo subtotal resection, cyst drainage, and Ommaya reservoir insertion	Visual deterioration in one eye Single hormone deficiency Hypothalamic dysfunction No cognitive/behavioral difficulties Surgical complication – shunt failure

**Fig. 2:**
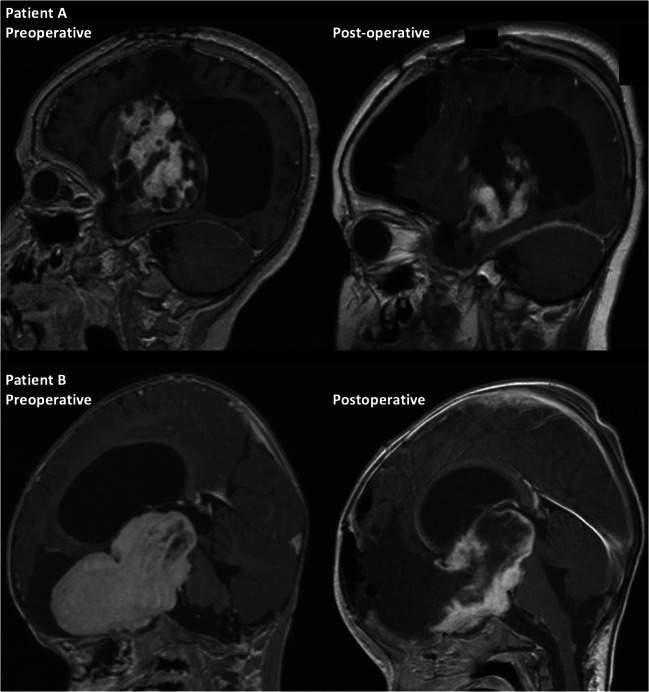
Examples of pre- and post-operative imaging following subtotal surgical resection**. P**re- and post-operative images of two patients (patient A and patient B) showing subtotal resection of an optic pathway glioma demonstrating conservative resection at the anterior skull base, and along the floor and posterior walls of the third ventricle where the hypothalamus is often indistinguishable from the tumor. Patient A also demonstrates drainage of a periventricular tumor cyst

An example of pre- and post-operative imaging following a subtotal resection of a grade I pilocytic optic pathway astrocytoma is presented in Fig. 2. In this case, the OPG was involving the hypothalamus, pituitary stalk, and encasing the internal carotid arteries. Therefore, a conservative debulking of the frontal component was undertaken in order to minimize post-operative deficits. The patient also had a ventriculoperitoneal shunt inserted for obstructive hydrocephalus and was treated with several courses of chemotherapy.

## Discussion

OPG is a complex and challenging disease. Although general consensus statements exist, there remains uncertainty around the optimal surgical strategy. The long-term efficacy of various treatments for newly diagnosed OPGs is also largely unknown. The timing and nature of the surgical approaches to OPGs have not been subjected to rigorous trial data, and ongoing studies are mainly focused on pharmaceutical interventions in recurrent/refractory disease, and to a lesser extent on diagnostics and assessment of social functioning [29]. Surgical options are broad and include biopsy, partial/subtotal resection, and radical resection, in addition to cerebrospinal fluid diversion for associated hydrocephalus [13]. Unlike pilocytic astrocytomas in non-eloquent locations, complete resection is rarely feasible due to the diffuse infiltrating nature of OPG and the proximity and involvement of eloquent brain structures. Due to the unpredictability of their behavior, and reported stability of a high proportion of OPGs, initial surveillance is often employed as an initial approach if there is no evidence of cerebrospinal fluid obstruction or visual deficit [28]. Most contemporaneous reports focus on the role of observation, chemotherapy, and radiotherapy with less focus on surgery. Published guidelines tend to recommend reservation of surgery for carefully selected cases where biopsy or debulking can be achieved with limited patient risk. It is important when deciding on therapeutic options to remain cognizant that these young patients often face a lifetime of morbidity relating to visual dysfunction, endocrinopathy, or any complications of treatment and that this can have long-term psychosocial consequences [30]. A summary of previously published guideline recommendations relating to surgical management of OPGs are presented in Table 5.

**Table 5:** Key published guidelines relating to surgical management of optic pathway glioma

Author and reference	Title	Summary of key recommendation/s
National Institute for Health and Clinical Excellence 2018 [31]	Brain tumors (primary) and brain metastases in adults (NG99)	- Consider active monitoring without a histological diagnosis, for lesions with radiological features typical of optic pathway glioma
International Society of Pediatric Oncology 2017 [32]	SIOP PODC Adapted treatment guidelines for low grade gliomas in low- and middle-income settings	- Observation of incompletely resected optic pathway gliomas, especially in NF1 patients, should be the rule
British Neuro-Oncology Society 2011 [33]	Guidelines on the diagnosis and management of optic pathway glioma (OPG)	- Surgery of any sort results in almost inevitable damage to the optic nerve or pathway and where imaging characteristics are classical biopsy cannot usually be justified - Where useful vision is maintained on the side of the tumor, it is hard to argue for debulking or resective surgery - In the context of failing or useless vision on one side, debulking or resective surgery may be appropriate to protect vision on the good side and potentially control hydrocephalus and delaying radiotherapy - Probably better to have a strategy of managing these patients on an individualized basis - Biopsy may be appropriate if imaging is atypical - CSF shunting and surgery to decompress tumor-related cysts will have a role in some patients - Debulking of large tumors is most likely to be valuable where continued growth is threatening or causing neurological deterioration - Radical or complete resections will be unusual in the region of the hypothalamus due to risks of causing post-operative hypothalamic syndrome
National Institute for Health and Clinical Excellence 2006 [34]	Improving outcomes for people with brain and other CNS tumors	- The majority of these patients will be managed initially by the neuroscience brain and other CNS tumors multidisciplinary team (MDT) - National tumor groups should be established and funded to standardize care for these patients Uncertainties surrounding treatment requires specialist input
Listernick et al 2007 [35]	Optic pathway gliomas in Neurofibromatosis-1: controversies and recommendations	- Surgical decompression limited to partial removal of the intraorbital optic nerve should be performed on NF1-associated optic nerve gliomas only for cosmetic purposes or to treat corneal exposure - Optic nerve gliomas without chiasmal involvement at initial presentation do not “grow backward” and extend into the chiasm, surgical removal of an optic nerve glioma, particularly the intracranial portion, to prevent “spread” to the chiasm is unnecessary - Hypothalamic or chiasmal gliomas occasionally may require surgical decompression, especially when hydrocephalus occurs due to third ventricular compression - Surgical biopsy is not generally useful for typical OPG in children with NF1, it may have some utility for NF1-associated OPGs with an unusual location or presentation

Unfortunately, a proportion of OPGs will grow, even with maximal chemo-radiotherapeutic treatment. As such, it is suggested that some OPGs may benefit from surgery although the exact timing and role is not universally accepted. Some view surgery as having no place in treatment, and others take a more interventional aggressive stance advocating it as up-front chemotherapy sparing or as rescue therapy. Perhaps unsurprisingly given the inherent challenges of surgical trials in a rare childhood condition, prospective comparative clinical trials that directly explore the effects of surgery, and how it is optimally delivered, are lacking. A number of articles have been written on the subject, and key references are summarized in Table 6.

**Table 6:** Key publications of surgical management of optic pathway glioma

Author and reference	Title	Population	Key finding/s and conclusion/s
Hidalgo et al. 2019 [13]	Long-term clinical and visual outcomes after surgical resection of pediatric pilocytic/pilomyxoid optic pathway gliomas	*n* = 83	- Approximately half of all children experience a long-term benefit from resection both as primary treatment and as a second-line therapy after failure of primary treatment. - Patients in whom chemotherapy failed have a retrieval rate similar to those treated upfront with surgery. Resection at the time of failure of the primary treatment - Primary surgery does not appear to have a significant benefit for children younger than 2 years or tumors with pilomyxoid features - There was an inherent bias of including only children who were referred and amenable to surgical intervention
Bin Abdulqader et al 2019 [36]	Endoscopic transnasal resection of optic pathway pilocytic astrocytoma	*n* = 4	- Authors reported that endoscopic endonasal approach provided a direct corridor to OPG with acceptable results in terms of tumor resection and visual outcomes
Liu et al 2018 [37]	Analysis of survival prognosis for children with symptomatic optic pathway gliomas who received surgery	*n* = 125	- Surgery is safe and feasible for children with large-volume OPGs, functional impairments, and obstructive hydrocephalus - Partial intratumor resection is recommended using neuronavigation guidance system
El Beltagy et al 2016 [38]	Treatment and outcome in 65 children with optic pathway gliomas	*n* = 65	- The initial role of surgery in newly developed OPG is biopsy for tissue diagnosis and relief of the hydrocephalus if present - More radical surgery may not be indicated as an initial therapy, but surgery may have a definite role in controlling progression of the disease upon failure of chemotherapy
Millward et al 2015 [39]	The role of early intra-operative MRI in partial resection of optic pathway/hypothalamic gliomas in children	*n* = 10	- Control of tumor progression was achieved in all patients without any surgically related mortality or morbidity. Seven patients had on table second-look surgery with significant further tumor resection following intraoperative MRI - Intraoperative MRI is a safe and useful additional tool, to be combined with advanced neuronavigation techniques for partial tumor resection
Goodden et al 2014 [28]	The role of surgery in optic pathway/hypothalamic gliomas in children	*n* = 42	- Surgery has a clear role for diagnosis, tumor control, and relief of mass effect. In particular, primary surgical debulking of tumor (without adjuvant therapy) is safe and effective
Sawamura et al 2008 [27]	Role of surgery for optic pathway/hypothalamic astrocytomas in children	*n* = 25	- The open biopsy offered no noteworthy benefit for the patients despite surgical risk and delay of chemotherapy - Surgical resection was not curative in any patient - The role of surgical intervention is restricted to bulk-reduction surgery only when it is inevitable, especially at relapse after chemotherapy
Leonard et al 2006 [40]	The role of surgical biopsy in the diagnosis of glioma in individuals with neurofibromatosis 1	*n* = 17	- Biopsy in NF1- associated gliomas with unusual clinical or radiographic presentations shows a high rate of tumors that are not classical pilocytic astrocytomas and may include higher grade tumors
Khafaga et al 2003 [41]	Optic gliomas: a retrospective analysis of 50 cases	*n* = 50	- Complete surgical resection is successful in tumors confined to an optic nerve. For disease that has progressed into or beyond the chiasm, total resection is not practical. Partial resection may provide symptomatic relief and will provide a tissue diagnosis - The correct balance of partial resection, surveillance, radiotherapy, and chemotherapy remains to be determined for this disease
Sutton et al 1995 [20]	Long-term outcome of hypothalamic/chiasmatic astrocytomas in children treated with conservative surgery	*n* = 33	- It is unlikely that any single modality will be the optimum treatment for all children with hypothalamic/chiasmatic astrocytoma
Tenny et al 1982 [42]	The neurosurgical management of optic glioma. Results in 104 patients	*n* = 104	- Unilateral resectable tumor should be removed, assuming vision is severely compromised, but a conservative approach should be taken in patients in whom vision is preserved and proptosis minimal - Tumor resection is advised to relieve obstructive hydrocephalus or for biopsy purposes - Posterior lesions should be treated by radiation therapy usually after surgical exploration if indicated

In terms of our surgical experience, we performed a total of 104 operations on 50 individuals during the study period. Many patients underwent several procedures. Our surgical strategy is summarized below, subclassified by operation type.

### Biopsy

Thirty-one biopsies were undertaken in a total of 30 patients, and 4 of these went on to have resective surgery later. While some centers advocate biopsy for all suspected OPGs, in our unit a biopsy is generally reserved for NF1 negative cases with radiologically uncertain pathology where the information may guide treatment decisions, particularly in terms of chemotherapy regimen choice. OPG is not a single disease and can include several distinct tumor subtypes as evidenced by our data on histological diagnosis (Table 1). In most cases, we favor stereotactic biopsy for these deep-seated lesions, particularly expansile Dodge 2 or 3 tumors. In purely intraconal tumors, biopsy under direct vision is more appropriate.

Our unit treats children with OPG as per the Children’s Cancer and Leukaemia Group (CCLG) pediatric low-grade glioma guidelines where standard first-line chemotherapy choice is typically carboplatin and vincristine (Packer regimen), vinblastine is second line, and bevacizumab and irinotecan are third line. This protocol was followed in all biopsy cases except one that was treated with temozolomide on the basis of high-grade glioma features and two patients with adverse reactions to carboplatin. Chemotherapeutic agent rechallenges were considered on an individual basis. Fourth line and subsequent treatments were more variable and included vinorelbine or TPCV combination treatment.

The rate of biopsies in our series has increased in recent years largely due to the availability of targeted inhibitors and emerging evidence of their efficacy in controlling these tumors [29]. The choice of molecular inhibitor, e.g., BRAF, MEK or FGFR depends on the underlying biology, and so biopsy is essential prior to commencing such specific agents in non-NF1 associated pediatric low-grade glioma. In this series, molecular testing permitted small molecule agents (dabrafenib and/or trametinib) to be used in 10 patients, all of whom had relapsed or progressive disease and had already received at least first line standard of care chemotherapy. Future trials, such as the LOGGIC study (low-grade glioma in children) run by the European Society for Paediatric Oncology (SIOPE), are planned that will compare upfront standard chemotherapy to targeted molecular therapy with trametinib.

### Resection—subtotal and gross total

The details of resected cases are presented in Table 4. A total of 17 patients had at least one resection, and gross total resection was achieved in 4 patients. This is the most controversial aspect of OPG management with strong proponents of both conservative and aggressive surgical approaches remaining. The consensus is currently shifted towards more conservative approaches because of the recognition of the significant harm that can be encountered in these cases, particularly by surgically exacerbated endocrine, visual, or hypothalamic dysfunction. OPGs are often diffusely infiltrative lesions, and the lack of clear margins makes complete resection challenging even when function can be preserved. Surgical experience is important when tackling this rare pathology. We propose that indications still exist for a more aggressive attempt at subtotal or gross total resection. In our experience, this is mainly indicated for lesions that are spatially divorced from the hypothalamic region, already associated with significant visual dysfunction, and show an aggressive local growth pattern with resulting mass effect. Classically this is the case when a single optic nerve is involved and causing progressive disfiguring proptosis or blindness. An alternative scenario is when mass effect is causing obstructive hydrocephalus and resection would potentially allow restoration of cerebrospinal fluid flow and avoidance of a shunt. In other scenarios, or less acute presentations, we generally prefer to defer surgery in these cases as long as clinically tolerated, and chemotherapy remains our first line in the majority of cases. Continued radiological or symptomatic progression despite multiple courses or lines of chemotherapy or biological therapy are also indications for resection surgery. It has previously been reported that decompression of the chiasm and the less involved optic nerve during tumor resection may improve vision in the less affected eye [8]. Our results support this position. Although total numbers were small, we did not find any difference in rates of cognitive dysfunction in the operated (including targeted resection) versus conservatively managed groups (77% v 83%). Rates of normal endocrine function were lower in patients who underwent resection (65%) versus those who underwent non-resective surgery (88%) or no surgery (80%). Unfortunately, it is not possible to determine causality and timing from our data (Table 3). Recurrence was defined as tumor regrowth following treatment. This occurred in 82% of resected cases and 23% of those who did not have surgery—likely reflecting the aggressive nature and prior failure of chemotherapeutic control of the cases on whom we operated.

The threshold for surgery in OPGs associated with a diagnosis of neurofibromatosis 1 should be set highly. These patients tend to have diffuse disease and poorer response to surgery; furthermore, their disease can spontaneously stabilize at an older age in some cases [1]. In our series, only one patient undergoing resection had a diagnosis of neurofibromatosis 1. In cases of chemotherapy failure, then the choice between radiotherapy and surgery should be made in a multidisciplinary setting. It is important to recognize that there may be technical challenges of surgery undertaken after radiotherapy and wound healing may be impaired [43]. Cautious surgery is also often required in the context of hemorrhagic OPGs. Although rare this has been well-described [44]. Acute hemorrhage within OPGs can lead to a rapid deterioration in neurological condition, with acute hydrocephalus, hypothalamic failure, and dystonia. Depending on their clinical condition, urgent CSF diversion, followed by cautious tumor mass reduction, evacuation of the hematoma, and chemotherapy has in our experience led to a satisfactory long-term outcome.

### Cyst drainage

This category includes both microscopic and endoscopic drainage of tumor cysts. The association of cysts with OPGs is an important aspect of their macroscopic pathology due to the potential for rapid volumetric changes and the option of cystic drainage/decompression. We performed 17 cyst drainages. Seven of these were primary procedures, and 10 were second- or third-line procedures. Management of cysts is challenging as they can be rapidly dynamic and often resistant to chemotherapy, and radiotherapy may also exacerbate cystic growth in some cases. Specific therapies to reduce cyst formation are required [45].

### Ommaya reservoir insertion

It is our experience that in the case of cysts causing mass effect, a single drainage episode may not be sufficient and substantial relief can be afforded by Ommaya insertion with repeated aspirations as necessary. The highly proteinaceous nature of the fluid in most of these cysts often limits the long-term effectiveness of this strategy. We inserted an Ommaya reservoir on 4 occasions in this series.

### Cerebrospinal fluid diversion

The association of OPG with hydrocephalus is well recognized. As previously discussed, some cases can be managed by tumor debulking or medical management to restore CSF flow. If this is unsuccessful, or if the hydrocephalus is acute, or if other surgery is deemed unfavorable, then direct CSF diversion may be required. In our series this required ventriculoperitoneal shunt (VPS) insertion on 22 occasions. In a single case, an endoscopic third ventriculostomy (ETV) was performed.

As this was a retrospective observational study, there was no pre-specified treatment strategy for our patients. Our approach is one of pragmatic avoidance of harm and can be summarized as follows:

1. Optic pathway glioma is a complex pathology and patient treatment is individually tailored. Where possible decisions should be made with expert multidisciplinary consensus. Treatment decisions should consider age, neurofibromatosis status, tumor location, visual, endocrine and cognitive function, previous therapy, and patient wishes.
2. If there is acute symptomatic hydrocephalus, then this requires surgical intervention. Debulking surgery may restore flow in some cases; otherwise, placement of a VP shunt will be appropriate in most cases. In more indolent cases, then a trial of chemotherapy may be appropriate with suitably close monitoring for response or progression of hydrocephalus.
3. Until recently a radiological diagnosis was sufficient in most cases and remains the case on the background of NF1. However, with the advent of biologically targeted therapies or if there is any diagnostic uncertainty, biopsy in OPG if it can be performed safely should be considered, and then a stereotactic biopsy of the tumor is appropriate.
4. If there is mass effect from the tumor causing neurological compromise, then surgical resection should be considered, particularly if chemotherapy has failed or radiotherapy is not an option (e.g., due to young age).
5. The extent of resection depends on the tumor location and the existing neurological deficit. The goal is to preserve or improve neurological function, and therefore new deficits should be aggressively avoided.
6. Total resection is considered where this can be achieved without causing new deficits (e.g., in patients who have already lost vision) and in whom tumor is demonstrating progression despite the maximum tolerated chemoradiotherapy.
7. In selected cases where a cyst is causing mass effect, then placement of an Ommaya reservoir for drainage may be appropriate.

There are many important questions that remain to be answered with regards OPG. These include those listed in Table 7.

**Table 7:** Remaining questions in the surgical management of optic pathway glioma

How can we predict a particular clinical course—including tumor stability or response to chemotherapy—so that we can select those who may require surgical intervention?	
How do surgical interventions alter the natural history?	
Which cases will benefit from surgical intervention?	
Which surgical approaches/interventions afford the most benefit (and how would those cases best be selected)?	
What is the optimal timing of a given surgery?	
What factors predict/determine surgical outcomes?	
Which emerging developments can improve surgical outcome?	

Answering these questions in the context of a rare heterogeneous pathology is highly challenging and is likely to require a coordinated multicenter approach with comprehensive prospective recording of common data elements.

### Study limitations

The main limitation in this study, as with most in OPGs, is the inherent heterogeneity of the condition. OPGs constitute a range of different pathological and molecular entities including NF1 status that profoundly impact the natural history of the disease and response to chemotherapy. Their location also varies widely as do the associated clinical deficits seen. As OPG is a rare disease, it is challenging to capture their full heterogeneity without diluting any series to the point of irrelevance. However, failure to recognize the inherent differences in this diversity will mask subgroup effects. The data presented here is retrospective and represents the experience of a single center over a long time period and with multiple surgeons. This introduces variability into the practice that limits direct correlation of interventions with outcomes. Due to the retrospective nature of this study, our report lacks detailed cognitive function assessment and quality of life measures. We present our personal experience but counsel caution in extrapolating data to the activity of other units with different populations or clinical service organization. The management of OPGs requires individualized treatment plans made in a multidisciplinary fashion with the patient and their family at the core of the process.

## Conclusion

OPGs are a debilitating childhood tumor that causes substantial suffering. As our understanding of this disease is incomplete, and there is often a chronic history with high treatment burden and impaired functional outcomes, we need to develop better evidence based management strategies [1, 46]. Here we present our experience in managing surgical aspects of this challenging and heterogeneous disease.

## Abbreviations

CCLG: Children’s Cancer and Leukaemia Group
ETV: Endoscopic third ventriculostomy
GOSH: Great Ormond Street Hospital
JPA: Juvenile pilocytic astrocytomas
LOGGIC: Low-grade glioma in children study
NF1: Neurofibromatosis type 1
OPG: Optic pathway glioma
PXA: Pilomyxoid astrocytomas
SIOPE: European Society for Paediatric Oncology
TPCV: Thioguanine, procarbazine, lomustine, vincristine
VPS: Ventriculoperitoneal shunt
WHO: World Health Organization
